# Transcriptome Analysis of HepG2 Cells Expressing ORF3 from Swine Hepatitis E Virus to Determine the Effects of ORF3 on Host Cells

**DOI:** 10.1155/2016/1648030

**Published:** 2016-08-28

**Authors:** Kailian Xu, Shiyu Guo, Tianjing Zhao, Huapei Zhu, Hanwei Jiao, Qiaoyun Shi, Feng Pang, Yaying Li, Guohua Li, Dongmei Peng, Xin Nie, Ying Cheng, Kebang Wu, Li Du, Ke Cui, Wenguang Zhang, Fengyang Wang

**Affiliations:** ^1^College of Agriculture, Hainan University, Hainan Key Lab of Tropical Animal Reproduction & Breeding and Epidemic Disease Research, Animal Genetic Engineering Key Lab of Haikou, Haikou 570228, China; ^2^Modern Agriculture Risk Warning and Prevention and Control Center of Hainan Province, Haikou 571100, China; ^3^College of Animal Science, Inner Mongolia Agricultural University, Inner Mongolia Key Laboratory of Animal Genetics, Breeding and Reproduction, Inner Mongolia, Hohhot 010018, China

## Abstract

Hepatitis E virus- (HEV-) mediated hepatitis has become a global public health problem. An important regulatory protein of HEV, ORF3, influences multiple signal pathways in host cells. In this study, to investigate the function of ORF3 from the swine form of HEV (SHEV), high-throughput RNA-Seq-based screening was performed to identify the differentially expressed genes in ORF3-expressing HepG2 cells. The results were validated with quantitative real-time PCR and gene ontology was employed to assign differentially expressed genes to functional categories. The results indicated that, in the established ORF3-expressing HepG2 cells, the mRNA levels of CLDN6, YLPM1, APOC3, NLRP1, SCARA3, FGA, FGG, FGB, and FREM1 were upregulated, whereas the mRNA levels of SLC2A3, DKK1, BPIFB2, and PTGR1 were downregulated. The deregulated expression of CLDN6 and FREM1 might contribute to changes in integral membrane protein and basement membrane protein expression, expression changes for NLRP1 might affect the apoptosis of HepG2 cells, and the altered expression of APOC3, SCARA3, and DKK1 may affect lipid metabolism in HepG2 cells. In conclusion, ORF3 plays a functional role in virus-cell interactions by affecting the expression of integral membrane protein and basement membrane proteins and by altering the process of apoptosis and lipid metabolism in host cells. These findings provide important insight into the pathogenic mechanism of HEV.

## 1. Introduction

Hepatitis E infection, caused by enterically transmitted hepatitis E virus (HEV), is a public health problem worldwide, particularly in developing countries such as China and India [1]. HEV infection is associated with a mortality rate of 0.2–1% in the general population, with an increased incidence and severity in pregnant women, in which mortality rates of 15–20% are observed [2–4]. As a zoonotic disease, swine infected with swine hepatitis E virus (SHEV) are the major reservoir of human HEV contamination [5, 6].

The HEV genome contains three open reading frames (ORFs), which encode ORF1, ORF2, and ORF3. ORF3 is a small molecular protein that influences multiple signal pathways in host cells [4]. In our previous study, the downregulation of microRNAs miR-221 and miR-222 in ORF3-expressing HEK 293 cells was observed, and miR-221 and miR-222 were found to directly regulate p27^kip1^. Our findings suggested that ORF3 might be involved in the proliferation of the host cells [7].

As one of the next-generation sequencing technologies, RNA-Seq can provide a complete snapshot of all of the transcripts present at a particular moment in the cell. RNA-Seq is superior to the oligonucleotide microarray approach that analyzes a selected number of previously defined transcripts. Based on RNA-Seq transcriptome analysis results and differential expression validation with quantitative real-time PCR (qRT-PCR), the differentially expressed genes (DEGs) of Huh-7 cells transfected with the HEV replicon were obtained. These included some innate immune response associated genes and some cell survival and metabolism associated genes; however, the functional roles of ORF3 were not elucidated [8]. In our study, RNA-Seq-based screening and further qRT-PCR validation were performed to identify the DEGs in ORF3-expressing HepG2 cells, and the DEGs identified were assigned functions by gene ontology. Our findings suggested that ORF3 functions by affecting the biological processes, cellular components, and molecular functions within the host cells.

## 2. Materials and Methods

### 2.1. Cell Lines and Plasmids

HepG2 cells were purchased from the Cell Bank of the Chinese Academy of Sciences (Shanghai, China) and were grown at 37°C in Dulbecco's minimum essential medium (DMEM) (Gibco BRL, Carlsbad, CA, USA) containing 10% heat-inactivated fetal bovine serum (FBS) (Gibco BRL), supplemented with penicillin (100 U/mL; Gibco BRL) and streptomycin (100 *μ*g/mL; Gibco BRL, USA). The recombinant plasmid, pEGFP-ORF3, which expresses EGFP-ORF3 fusion protein, was constructed in our previous study [5].

### 2.2. Preparation of Recombinant Lentivirus

The upstream primer (5′-GCGGCGTTAATTAAGCCACCATGGCGATGCCACCATGCG-3′) containing a* Pac*I site and the downstream primer (5′-ATTATTGGCGCGCCTCAGCGGCGAAGCCCCAGCT-3′) containing an* Asc*I site were used to amplify the ORF3 fragment from pEGFP-ORF3. The obtained ORF3 fragment was ligated into the lentiviral vector, pLenti6.3-MCS-IRES-GFP, and then digested with* Pac*I and* Asc*I. The recombinant lentivirus was designated pLenti6.3-ORF3-IRES-EGFP. The recombinant lentivirus was prepared as previously described and the titers of the recombinant lentivirus were determined [7]. pLenti6.3-MCS-IRES-GFP was also used for the preparation of recombinant lentivirus, and this was used as a negative control in the experiments.

### 2.3. Establishment of SHEV ORF3-Expressing HepG2 Cells

As previously described, HepG2 cells were infected with the recombinant lentivirus at a multiplicity of infection (MOI) of 10 [5]. The expression of enhanced green fluorescence protein (EGFP) was observed by fluorescence microscopy (X71; Olympus, Tokyo, Japan). The stable cell lines were obtained as previously described [5].

### 2.4. Flow Cytometry Analysis

As previously described, FACSCalibur flow cytometer (Becton Dickinson, San Jose, CA, USA) was used to determine the percentage of fluorescent cells population and the meant fluorescent intensity of the stable cells lines [5].

### 2.5. Western Blot Analysis

The total protein from ORF3-expressing HepG2 cells, EGFP (only)-expressing HepG2 cells, and HepG2 cells was harvested. SDS-PAGE and western blot analysis were performed as previously described [5]. The primary antibodies used were rabbit polyclonal antibody against ORF3, which was produced as described in our previous study [5], and rabbit polyclonal anti-GAPDH antibody (Cell Signaling Technology, Beverly, MA, USA). The secondary antibody was HRP-labeled goat anti-rabbit IgG (Santa Cruz Biotechnology, Santa Cruz, CA, USA).

### 2.6. mRNA Library Construction and Sequencing

Following the manufacturer's procedure, TRIzol reagent (Invitrogen, Carlsbad, CA, USA) was used to extract the total RNA from ORF3-expressing HepG2 cells, EGFP (only)-expressing HepG2 cells, and HepG2 cells. The quantity and purity of the total RNA were analyzed using Bioanalyzer 2100 and the RNA 6000 Nano LabChip Kit (Agilent Technologies, Santa Clara, CA, USA).

Next, 10 *μ*g of RNA from specific cell lines (ORF3-expressing HepG2 cells, EGFP (only)-expressing HepG2 cells, and HepG2 cells) was exposed to poly-T oligoattached magnetic beads (Invitrogen, Carlsbad, CA, USA) to isolate poly(A) mRNA. Following purification, the mRNA was fragmented into small pieces using fragmentation buffer and the cleaved RNA fragments were reverse-transcribed to construct a cDNA library using the mRNA-Seq sample preparation kit (Illumina, San Diego, CA, USA), according to the manufacturer's instructions. Then, paired-end sequencing was performed on an Illumina 2000/2500 sequence platform (LC Sciences, Houston, TX, USA), following the manufacturer's protocol.

### 2.7. Mapping, Normalization, and Calculation of the RPKM

Clean reads were obtained by removing the low quality reads from the raw reads. The quality of the reads was classified according to the following criteria: (1) containing sequencing adaptors, (2) ratio of *N* (without valid base information) above 5%; and (3) ratio of nucleotides [*Q* value (quality score) is lower than 10] above 20%. Then, as previously described, clean reads from specific cell lines were aligned to the genome database UCSC (http://genome.ucsc.edu/) using the Tophat package [9].

Based on the results of Tophat, the fragment per kilobase of exon per million fragments mapped (FPKM) value was used to normalize the number of fragments, as previously described. Cufflinks were used to de novo assemble the transcriptome and comerge and annotate the sequence fragments. The DEGs, their corresponding attributes, fold changes (in log_2_ scale), *p* values, and FDR (false discovery rate corrected *p* values) were obtained [10, 11]. The significance of the gene expression difference was determined as “yes” if the false discovery rate (*Q* value) was <0.05. Only the comparisons with *Q* value less than 0.01 and a status marked as “OK” in the Cuffdiff output were regarded as showing differential expression [12].

### 2.8. Gene Ontology (GO) of DEGs

As previously described, GO was performed to analyze the DEGs [13]. GO terms with *p* < 0.05 were considered significantly enriched among the DEGs [9].

### 2.9. qRT-PCR for Differential Expression Validation

To validate the differential expression of genes, the specific primers were designed and qRT-PCR was performed as previously described [7].

### 2.10. Statistical Analysis

The statistical significance of the differences between the data from the experimental groups and the control was analyzed using Student's *t*-test and one-way ANOVA. *p* < 0.05 was considered to represent significant differences, and *p* < 0.01 was considered as highly different [7].

## 3. Results and Discussion

### 3.1. Identification of SHEV ORF3-Expressing HepG2 Cells

**Figure 1:**
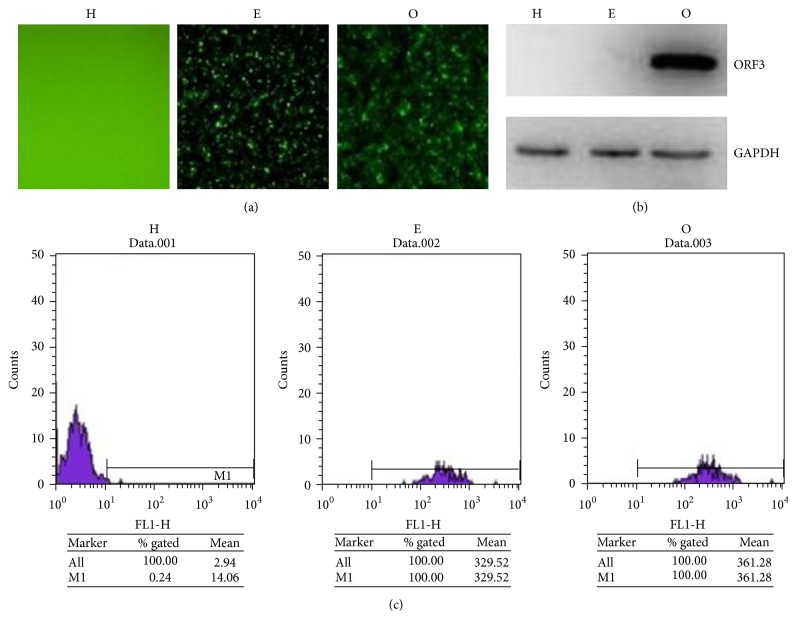
Characterization of ORF3-expressing HepG2 cells. (a) Fluorescence observation of H, E, and O cells. (b) Western blot results indicated that ORF3 protein was expressed in O cells. (c) Flow cytometry analysis results indicated that EGFP protein was expressed in O cells and E cells.

After the recombinant lentivirus vector, pLenti6.3-ORF3-IRES-EGFP, had been constructed and confirmed with DNA sequencing, recombinant lentivirus carrying ORF3 was prepared, titrated, and used to infect HepG2 cells. After blasticidin selection, ORF3-expressing HepG2 cells were obtained and designated as O, and EGFP (only)-expressing HepG2 cells were obtained and designated as E. HepG2 cells were used as the black control and designated as H. The expression of EGFP protein was observed in O and E cells (Figures 1(a) and 1(c)). Western blotting results revealed that there was a specific band at the expected molecular weight for ORF3 protein in O cells; however, no expression of ORF3 was detected in E and H cells (Figure 1(b)).

### 3.2. Sequencing and Mapping of the SHEV ORF3-Expressing HepG2 Cell Transcriptome

Using the Illumina paired-end RNA-Seq approach, the cDNA libraries of H, E, and O cells were sequenced. The results were uploaded into NCBI Sequence Read Archive (SRA) (accession number: SRP073936). The average insert size for the paired-end libraries was 300 bp (±50 bp). In total, 132,757,090 paired-end reads of 2 × 100 bp length were acquired. The total read length of the three samples was 16.59 gigabases (Gb). After removing the low quality reads from the raw reads, a total of 16.42 Gbp of cleaned, paired-end reads were produced, with a Q20 of over 90% (Table 1).

Alignment of the sequence reads against the reference genome yielded about 70% aligned reads across the three samples, for which the ratio of pair reads is about 30% and the ratio of unique map was about 70% and of which about 98% were located within annotated exons. Multiposition matched reads (<10%) were excluded from further analyses. The distribution of the density of the sequence was normal. These data satisfied the requirements of further gene expression level analyses.

### 3.3. Differential Expression Analysis

**Figure 2:**
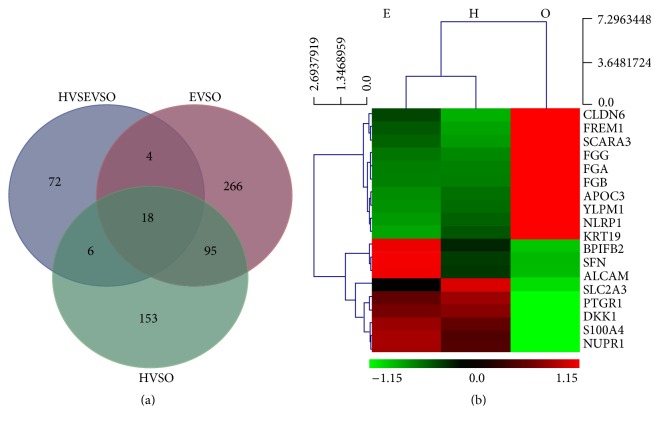
The DEGs obtained from H, E, and O cells by RNA-Seq. (a) The Venn diagrams indicated that the number of the significantly DEGs in O cells was 18. (b) Heat-maps indicated that, compared with that in H and E cells, in O cells, the mRNA levels of CLDN6, FREM1, SCARA3, FGG, FGA, FGB, APOC3, YLPMA1, and NLRP1 were upregulated, while KRT19, BPIFB2, SFN, ALCAM, SLC2A3, PTGR1, DKK1, S100A4, and NUPR1 were downregulated. Red indicates upregulation and green indicates downregulation.

Visualization of the data in Venn diagrams indicated that the number of DEGs in O cells was 18. In O cells, the mRNA levels of claudin-6 (CLDN6), FRAS1-related extracellular matrix 1 (FREM1), scavenger receptor class A member 3 (SCARA3), fibrinogen (FGG), fibrinogen alpha (FGA), fibrinogen beta (FGB), apolipoprotein C3 (APOC3), YLP motif-containing protein 1 (YLPM1), and nucleotide-binding oligomerization domain-like receptor with pyrin domain protein 1 (NLRP1) were upregulated, while the mRNA levels of cytokeratin 19 (KRT19), BPI fold containing family B, member 2 (BPIFB2), sulforaphane (SFN), activated leukocyte cell adhesion molecule (ALCAM), solute carrier family 22 member 3 (SLC2A3), prostaglandin reductase 1 (PTGR1), Dickkopf-related protein 1 (DKK1), S100 calcium binding protein A4 (S100A4), and nuclear protein 1 (NUPR1) were downregulated (Figures 2(a) and 2(b), Table 2).

**Figure 3:**
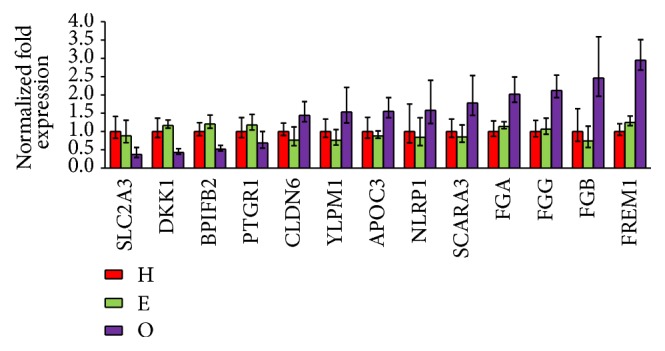
Validation of the RNA-Seq data by qRT-qPCR. The results confirmed the upregulation of the mRNA levels of CLDN6, YLPM1, APOC3, NLRP1, SCARA3, FGA, FGG, FGB, and FREM1 and the downregulation of the mRNA levels of SLC2A3, DKK1, BPIFB2, and PTGR1.

To further confirm the RNA-Seq data, specific primers were designed (Table 3), and the qRT-PCR was performed using GAPDH as an internal control. The results confirmed that the mRNA levels of CLDN6, YLPM1, APOC3, NLRP1, SCARA3, FGA, FGG, FGB, and FREM1 were upregulated and the mRNA levels of SLC2A3, DKK1, BPIFB2, and PTGR1 were downregulated (Figure 3).

### 3.4. Assignment of DEGs

**Figure 4:**
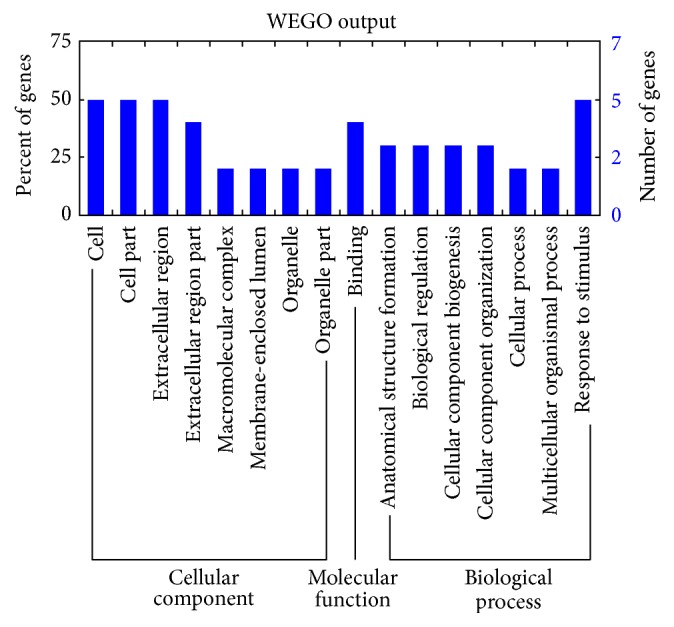
GO classifications of the DEGs in O cells.

The 13 validated DEGs in O cells were assigned into the following categories: biological process, cellular components, and molecular function (*p* < 0.05) (Figure 4; see S1 in Supplementary Material available online at http://dx.doi.org/10.1155/2016/1648030). Among them, FGG, FGA, and FGB were assigned into all 25 GO cases for category biological processes, all 18 GO cases for cellular components, and 4 GO cases for molecular function (S1).

The cellular component category includes membranes, organelles, and proteins. In O cells, two validated DEGs, CLDN6 and FREM1, were assigned into the cellular component category (S1). CLDN6 encodes an integral membrane protein that is one of the entry cofactors for hepatitis C virus, which was assigned into GO: 0005886-plasma membrane [14]. FREM1 encodes a basement membrane protein, which was assigned into GO: 0044421-extracellular region part and GO: 0005576-extracellular region [15]. These results suggested that the expression of ORF3 induced the deregulated expression of two cellular components.

Biological processes include many chemical reactions and other events that result in chemical transformation including metabolism and homeostasis. In O cells, NLRP1 was assigned into the GO category of biological processes (S1). The NLRP1 gene encodes a member of the Ced-4 family of apoptosis proteins. NLRP3/NLRP1 inflammasome-mediated caspase-1 activation with subsequent IL-1 secretion is essential for the subsequent bifurcation to downregulated proinflammatory cytokines and upregulated bacterial killing [16], and this gene was assigned into two GO cases within the category of biological processes (GO: 0006950 and GO: 0050896-response to stress and stimulus, resp.). These results suggested that the expression of ORF3 affects the apoptosis of HepG2 cells.

The liver is the central regulatory organ of lipid pathways. APOC3 specifically modulates the metabolism of triglyceride-rich lipoproteins and may contribute to the development of hyperlipidemia and other lipoprotein abnormalities in humans [17–19]. In our study, APOC3 was one of the 13 validated DEGs in O cells. APOC3 not only was assigned into three GO cases within the category “cellular components” (GO: 0044421-extracellular region part; GO: 0005576-extracellular region; GO: 0005615-extracellular space) and five GO cases of biological process (GO: 0065003-macromolecular complex assembly; GO: 0043933-macromolecular complex subunit organization; GO: 0022607-cellular component assembly; GO: 0044085-cellular component biogenesis; GO: 0065008-regulation of biological quality), but was also assigned into two GO cases within the category “molecular function” (GO: 0005102-receptor binding and GO: 0070325-lipoprotein receptor binding) (S1).

Interestingly, SCARA3 and DKK1 are also related to the lipid metabolism. As a member of the scavenger receptor family, SCARA3 protects cells by scavenging reactive oxygen species and other harmful products of oxidation [20]. SCARA3 binds to polyanionic ligands, which are an important source of fatty acids for macrophages [21]. SCARA3 was assigned into three GO cases within the category “biological processes” (GO: 0042221-response to chemical stimulus; GO: 0006950-response to stress; GO: 0050896-response to stimulus) (S1).

DKK1, a canonical Wnt/*β*-catenin pathway antagonist, is closely associated with adipogenesis [22]. DKK1 regulates certain aspects of placental lipid metabolism through the WNT signaling pathway [23]. DKK1 not only was assigned into two GO cases within the category “cellular components” (GO: 0005576-extracellular region; GO: 0005886-plasma membrane), but also was assigned into two GO cases within the category “molecular function” (GO: 0005102-receptor binding; GO: 0070325-lipoprotein receptor binding), as seen for APOC3 (S1).

From these data, it can be concluded that the expression of ORF3 induces the upregulation of the mRNA levels of CLDN6, YLPM1, APOC3, NLRP1, SCARA3, FGA, FGG, FGB, and FREM1 and the downregulation of the mRNA levels of SLC2A3, DKK1, BPIFB2, and PTGR1. Among these changes, the deregulated expression of CLDN6, APOC3, NLRP1, SCARA3, FGA, FGG, FGB, FREM1, SLC2A3, DKK1, and PTGR1 might contribute to the deregulation of integral membrane protein and basement membrane protein and affect the apoptosis and the lipid metabolism of HepG2 cells.

In a recent study, the RNA-Seq approach was used to explore the cellular pathway alterations during virus infection. Changes in the transcriptomes of primary bovine cells following infection with either wild type Schmallenberg virus (SBV) or SBV with a mutant lacking the nonstructural protein NSs (SBVdelNSs) were analyzed. The results suggested that nonstructural protein not only was effective in shutting down genes of the host innate immune system, but also affected a number of possible antiviral factors [24]. Because of the lack of a cell culture system and a suitable animal model, the pathogenesis of hepatitis E is poorly understood. In this study, HepG2 cells, which are a suitable in vitro model system for the study of HEV, were used as the target cells for ORF3 overexpression. RNA-Seq-based screening and further qRT-PCR validation were performed to identify the DEGs in ORF3-expressing HepG2 cells.

Correlation analysis results between the RNA-Seq and qRT-PCR data indicated that 13 of the 18 DEGs detected by RNA-Seq in O cells were validated by qRT-PCR. These results indicated that the experimental approach was effective. The five nonvalidated DEGs may have been the false-positive results generated by RNA-Seq.

As a standardized gene function classification system, GO describes the properties of genes and their products. In our study, the Database for Annotation, Visualization and Integrated Discovery (DAVID) software was used to obtain the GO ID, and Web Gene Ontology Annotation Plot (WEGO) software was used to plot the GO annotation results (http://wego.genomics.org.cn/cgi-bin/wego/index.pl).

The liver is the central regulatory organ of lipid pathways. Our findings confirmed that HEV infection causes alterations in lipid metabolism. Among 13 validated DEGs, APOC3, SCARA3, and DKK1 played a role in lipid metabolism. In HepG2 cells, the expression of ORF3 causes the deregulation of lipid metabolism, potentially resulting in cell injury.

The expression of ORF3 also resulted in the deregulation of CLDN6, NLRP1, FGA, FGG, FGB, FREM1, SLC2A3, and PTGR1, potentially resulting in cell injury as a result of changes in biological processes, cellular components, and/or the molecular function of HepG2 cells.

To our knowledge, this is the first report of the altered expression of CLDN6, YLPM1, APOC3, NLRP1, SCARA3, FGA, FGG, FGB, FREM1, SLC2A3, DKK1, BPIFB2, and PTGR1 in HEV-infected cells. Our findings provide insight into the critical events that take place during HEV infection.

## 4. Conclusions

ORF3 protein is a key regulatory protein of SHEV. Here, for the first time, we report the upregulation of the mRNA levels of CLDN6, YLPM1, APOC3, NLRP1, SCARA3, FGA, FGG, FGB, and FREM1 and the downregulation of the mRNA levels of SLC2A3, DKK1, BPIFB2, and PTGR1 in the established ORF3-expressing HepG2 cells. The deregulated expression of these 13 genes may lead to changes in the deregulation of integral membrane and basement membrane proteins and may affect the processes of lipid metabolism and apoptosis in human cells. These findings provide insight into the infection processes mediated by HEV and may be valuable in the development of future therapeutic strategies.

## Supplementary Material

The Supplementary Material meant the specific pathways of genes.

## Figures and Tables

**Table 1:** Numbers of reads in the reference genome.

Sample	E	H	O
Valid reads	46,413,284	45,043,398	39,935,156
Mapped reads	32,068,726 (69.09%)	29,911,216 (66.41%)	27,147,943 (67.98%)
Unique mapped reads	31,520,409 (67.91%)	29,386,695 (65.24%)	26,670,680 (66.78%)
Multimapped reads	548,317 (1.18%)	524,521 (1.16%)	477,263 (1.20%)
PE mapped reads	13,909,419 (29.97%)	13,120,894 (29.13%)	11,735,308 (29.39%)
Exon	98.27%	98.05%	97.78%

**Table 2:** Genes found to be significantly differentially expressed between cell types.

Gene short name	*p* value	E	H	O
		FPKM	FPKM	FPKM
FGG	2.252*E* − 37	205.24	190.90	465.10
FGB	1.2802*E* − 10	43.87	44.37	110.70
FGA	2.6364*E* − 24	147.94	145.66	328.38
APOC3	1.71*E* − 43	249.10	272.37	584.34
SLC2A3	1.3633*E* − 31	522.47	569.07	249.24
DKK1	2.97*E* − 44	781.57	719.76	320.23
KRT19	3.1275*E* − 46	415.28	205.75	83.07
BPIFB2	5.7055*E* − 35	295.48	128.29	59.84
CLDN6	5.7466*E* − 07	36.04	24.00	72.68
SFN	0.000023461	30.45	12.06	3.77
FREM1	0.000048529	18.29	13.07	42.37
ALCAM	0.00010227	10.00	18.79	0.62
YLPM1	0.0010757	19.50	21.74	44.22
SCARA3	0.0011159	2.53	1.08	12.00
NLRP1	0.0078786	1.51	3.31	10.90
S100A4	0.0079767	17.17	14.16	2.80
PTGR1	0.010836	30.77	31.65	12.99
NUPR1	0.022976	16.63	14.24	3.92

FPKM: fragment per kilobase of exon per million mapped fragments.

**Table 3:** Primers for qRT-PCR validation.

Gene short name	Forward primer sequence 5′-3′	Reverse primer sequence 5′-3′
KRT19	GGTCATGGCCGAGCAGAA	TTCAGTCCGGCTGGTGAAC
DKK1	AGAAAAGGCTCTCATGGACTAGAAAT	CCGGCAAGACAGACCTTCTC
APOC3	TGGCTGCCTGAGACCTCAAT	AGGAGCTCGCAGGATGGATA
FGG	TGGTTGGTGGATGAACAAGTGT	TGCCACCTTGGTAATAAACTCCAT
PIFB2	CTGGATGTGGTAGTGAACTTGAGACT	ACGTGGTCCCCTGAAGCTT
SLC2A3	GGCACACGGTGCAGATAGATC	GCAGGCTCGATGCTGTTCAT
FGA	CAGATGAGGCCGGAAGTGA	GATTTAGCATGGCCTCTCTTGGT
FGB	CAGCCGGTAATGCCCTCAT	TGTGAATGGTCATGGTCCTGTT
CLDN6	CTTGGATGATGGAGCCAAAGA	TGGCTTCTAAGATGGGCATGT
SFN	GCAGGCCGAACGCTATGA	TCCACGGCGCCTTTCA
FREM1	ACCTGGGCAACCTTGTAACTGTA	TGGTCGTTCAAACCTATCCAAA
ALCAM	CAATGCCCCAAACTTTCTCATAA	TGTCCCCAATCTTCACAAGCT
YLPM1	GGAAACTGCACCTCGTCACA	GCAGCATCTTGCAGCAAAGA
SCARA3	CCCTGAGAAGTTCAACATTTATTTCTT	GGGCAGAGGCAAGGATGAAT
NLRP1	CCCTCTATCGGCGTCTATCTGT	GCTCTTACCGTCTCTTATTCAGCAT
S100A4	CGCCAGTGGGCACTTTTTT	CAGCATCAAGCACGTGTCTGA
PTGR1	AAGAAATTTGGAAGGATTGCCATA	GAAGTGGGCCGGTTCTGTTA
NUPR1	GGGTGGCAGCAACAATAAATAGA	GGATGAACACACACCCAAGCT

## Abbreviations

HEV: Hepatitis E virus
SHEV: Swine hepatitis E virus
ORF3: Open reading frame 3
qRT-PCR: Quantitative real-time RT-PCR
GO: Gene ontology
DEGs: Differentially expressed genes
NGS: Next-generation sequencing
DMEM: Dulbecco's minimum essential medium
FBS: Heat-inactivated fetal bovine serum
MOI: Multiplicity of infection
FCM: Flow cytometry
SDS-PAGE: Sodium dodecyl sulfate polyacrylamide gel electropheresis
EGFP: Enhanced green fluorescent protein
HRP: Horseradish peroxidase
ANOVA: Analysis of variance
CLDN6: Claudin-6
APOC3: Apolipoprotein C3
NLRP1: Nucleotide-binding oligomerization domain-like receptor with pyrin domain protein 1
FREM1: FRAS1-related extracellular matrix 1
SCARA3: Scavenger receptor class A member 3
FGG: Fibrinogen
FGA: Fibrinogen alpha
FGB: Fibrinogen beta
YLPM1: YLP motif-containing protein 1
KRT19: Cytokeratin 19
BPIFB2: BPI fold containing family B, member 2
SFN: Sulforaphane
ALCAM: Activated leukocyte cell adhesion molecule
SLC2A3: Solute carrier family 22 member 3
PTGR1: Prostaglandin reductase 1
DKK1: Dickkopf-related protein 1
S100A4: S100 calcium binding protein A4
NUPR1: Nuclear protein 1
FPKM: Fragment per kilobase of exon model per million mapped fragments
DAVID: Database for Annotation, Visualization and Integrated Discovery
WEGO: Web Gene Ontology Annotation Plot
SBV: Schmallenberg virus.

## References

[1] Sayed I. M., Vercouter A.-S., Abdelwahab S. F., Vercauteren K., Meuleman P. (2015). Is hepatitis E virus an emerging problem in industrialized countries?. *Hepatology*.

[2] Khuroo M. S., Teli M. R., Skidmore S., Sofi M. A., Khuroo M. I. (1981). Incidence and severity of viral hepatitis in pregnancy. *The American Journal of Medicine*.

[3] Navaneethan U., Al Mohajer M., Shata M. T. (2008). Hepatitis E and pregnancy: understanding the pathogenesis. *Liver International*.

[4] Chandra V., Taneja S., Kalia M., Jameel S. (2008). Molecular biology and pathogenesis of hepatitis E virus. *Journal of Biosciences*.

[5] Liu T., Lei M., Jiao H. (2011). RNA interference induces effective inhibition of mRNA accumulation and protein expression of SHEV ORF3 gene in vitro. *Current Microbiology*.

[6] Rogée S., Le Gall M., Chafey P. (2015). Quantitative proteomics identifies host factors modulated during acute hepatitis E virus infection in the swine model. *Journal of Virology*.

[7] Cheng Y., Du L., Shi Q. (2013). Identification of miR-221 and -222 as important regulators in genotype IV swine hepatitis e virus ORF3-expressing HEK 293 cells. *Virus Genes*.

[8] Jagya N., Varma S. P. K., Thakral D., Joshi P., Durgapal H., Panda S. K. (2014). RNA-Seq based transcriptome analysis of hepatitis E virus (HEV) and hepatitis B virus (HBV) replicon transfected Huh-7 cells. *PLoS ONE*.

[9] Cui X., Hou Y., Yang S. (2014). Transcriptional profiling of mammary gland in Holstein cows with extremely different milk protein and fat percentage using RNA sequencing. *BMC Genomics*.

[10] Trapnell C., Williams B. A., Pertea G. (2010). Transcript assembly and quantification by RNA-Seq reveals unannotated transcripts and isoform switching during cell differentiation. *Nature Biotechnology*.

[11] Mortazavi A., Williams B. A., McCue K., Schaeffer L., Wold B. (2008). Mapping and quantifying mammalian transcriptomes by RNA-Seq. *Nature Methods*.

[12] Rapaport F., Khanin R., Liang Y. (2013). Comprehensive evaluation of differential gene expression analysis methods for RNA-seq data. *Genome Biology*.

[13] Young M. D., Wakefield M. J., Smyth G. K., Oshlack A. (2010). Gene ontology analysis for RNA-seq: accounting for selection bias. *Genome Biology*.

[14] Arabzadeh A., Troy T.-C., Turksen K. (2006). Role of the Cldn6 cytoplasmic tail domain in membrane targeting and epidermal differentiation in vivo. *Molecular and Cellular Biology*.

[15] Petrou P., Pavlakis E., Dalezios Y., Chalepakis G. (2007). Basement membrane localization of Frem3 is independent of the Fras1/Frem1/Frem2 protein complex within the sublamina densa. *Matrix Biology*.

[16] Hedl M., Abraham C. (2013). NLRP1 and NLRP3 inflammasomes are essential for distinct outcomes of decreased cytokines but enhanced bacterial killing upon chronic Nod2 stimulation. *American Journal of Physiology—Gastrointestinal and Liver Physiology*.

[17] Hofker M. H. (2010). APOC3 null mutation affects lipoprotein profile. APOC3 deficiency: from mice to man. *European Journal of Human Genetics*.

[18] Pollin T. I., Damcott C. M., Shen H. (2008). A null mutation in human *APOC3* confers a favorable plasma lipid profile and apparent cardioprotection. *Science*.

[19] Jong M. C., Havekes L. M. (2000). Insights into apolipoprotein C metabolism from transgenic and gene-targeted mice. *International Journal of Tissue Reactions*.

[20] Han H.-J., Tokino T., Nakamura Y. (1998). CSR, a scavenger receptor-like protein with a protective role against cellular damage caused by UV irradiation and oxidative stress. *Human Molecular Genetics*.

[21] Murphy J. E., Tedbury P. R., Homer-Vanniasinkam S., Walker J. H., Ponnambalam S. (2005). Biochemistry and cell biology of mammalian scavenger receptors. *Atherosclerosis*.

[22] Zhang Y., Ge C., Wang L. (2015). Induction of DKK1 by ox-LDL negatively regulates intracellular lipid accumulation in macrophages. *FEBS Letters*.

[23] Strakovsky R. S., Pan Y.-X. (2012). A decrease in DKK1, a WNT inhibitor, contributes to placental lipid accumulation in an obesity-prone rat model. *Biology of Reproduction*.

[24] Blomström A.-L., Gu Q., Barry G. (2015). Transcriptome analysis reveals the host response to Schmallenberg virus in bovine cells and antagonistic effects of the NSs protein. *BMC Genomics*.

